# Proton transport from the antimatter factory of CERN

**DOI:** 10.1038/s41586-025-08926-y

**Published:** 2025-05-14

**Authors:** M. Leonhardt, D. Schweitzer, F. Abbass, K. K. Anjum, B. Arndt, S. Erlewein, S. Endoh, P. Geissler, T. Imamura, J. I. Jäger, B. M. Latacz, P. Micke, F. Voelksen, H. Yildiz, K. Blaum, J. A. Devlin, Y. Matsuda, C. Ospelkaus, W. Quint, A. Soter, J. Walz, Y. Yamazaki, S. Ulmer, C. Smorra

**Affiliations:** 1 Institut für Experimentalphysik, Heinrich Heine Universität Düsseldorf, Düsseldorf, Germany; 2 Ulmer Fundamental Symmetries Laboratory, RIKEN, Saitama, Japan; 3 Institut für Physik, Johannes Gutenberg-Universität, Mainz, Germany; 4 GSI-Helmholtzzentrum für Schwerionenforschung, Darmstadt, Germany; 5 Max-Planck-Institut für Kernphysik, Heidelberg, Germany; 6 CERN, Geneva, Switzerland; 7 Graduate School of Arts and Sciences, University of Tokyo, Tokyo, Japan; 8 Institut für Quantenoptik, Leibniz Universität, Hannover, Germany; 9 Physikalisch-Technische Bundesanstalt, Braunschweig, Germany; 10 Imperial College London, London, UK; 11 ETH Zürich, Zürich, Switzerland; 12 Helmholtz-Institut Mainz, Mainz, Germany; 13 Present Address: Friedrich-Schiller-Universität Jena, Jena, Germany

**Keywords:** Physics, Techniques and instrumentation

## Abstract

Precision measurements using low-energy antiprotons, exclusively available at the antimatter factory (AMF) of CERN^1^, offer stringent tests of charge–parity–time (CPT) invariance, which is a fundamental symmetry in the Standard Model of particle physics^2^. These tests have been realized, for example, in antiprotonic helium^3^ and antihydrogen^4^. In our cryogenic Penning-trap experiments^5^, we measure the magnetic moments^6,7^ and charge-to-mass ratios of protons and antiprotons and now provide the most precise test of CPT invariance in the baryon sector^8^. Our experiments are limited by magnetic field fluctuations imposed by the decelerators in the AMF; therefore, we are advancing the relocation of antiprotons to dedicated precision laboratories. Here we present the successful transport of a trapped proton cloud from the AMF using BASE-STEP^9^—a transportable, superconducting, autonomous and open Penning-trap system that can distribute antiprotons into other experiments. We transferred the trapped protons from our experimental area at the AMF onto a truck and transported them across the Meyrin site of CERN, demonstrating autonomous operation without external power for 4 h and loss-free proton relocation. We thereby confirm the feasibility of transferring particles into low-noise laboratories in the vicinity of the AMF and of using a power generator on the truck^10^ to reach laboratories throughout Europe. This marks the potential start of a new era in precision antimatter research, enabling low-noise measurements of antiprotons, the charged antimatter ions ${\bar{{\rm{H}}}}^{+}$^11^ and ${\bar{{\rm{H}}}}_{2}^{-}$ (ref. ^12^), and other accelerator-produced ions, such as hydrogen-like lead or uranium ions^13,14^.

## Abstract (web summary)

The successful transport of a trapped proton cloud from the antimatter factory of CERN using a transportable, superconducting, autonomous and open Penning-trap system that can distribute antiprotons into other experiments is reported.

## Main

Precision measurements in low-energy quantum systems are used to explore the limits of the Standard Model of particle physics (SM)^15^, providing an opportunity to understand the nature of dark matter^16^ and the baryon asymmetry in the universe^17^. This approach motivates tests that compare conjugate matter–antimatter systems to examine the fundamental symmetries of the SM. Results of precision measurements on antiprotonic systems at low energies, including antiprotonic helium, antihydrogen and single trapped antiprotons, have been obtained recently, providing stringent tests of CPT invariance^3,4,6,8^. Moreover, the recent release of antihydrogen atoms from a vertical magnetic trap has provided direct and model-independent constraints on an antimatter-based test of the weak equivalence principle^18^. All these measurements are, without exception, performed inside the AMF hall of CERN. At the current level of measurement resolution, no deviations from established theory have been observed. However, these deviations could emerge with more sensitive measurements, highlighting the need for improved measurement precision.

Once a certain level of precision has been achieved in a quantum system, it becomes necessary to reduce the influence of external disturbance. Our measurements of spin-precession *ν*_L_ = *gqB*_0_/(4π*m*) and cyclotron frequencies *ν*_c_ = *qB*_0_/(2π*m*), carried out with single protons and antiprotons in cryogenic Penning-trap systems, give access to the charge-to-mass ratios *q*/*m* (ref. ^8^) and the *g*-factors of the particles^6,7^, with *g* defining their magnetic moments. These measurements are extremely sensitive to external magnetic field noise because the measured frequencies in the artificial exotic geonium atom—formed by the trap and the particle—scale directly with the magnetic field *B*_0_. Our recent comparison of the proton–antiproton charge-to-mass ratio at a fractional precision of 1.6 × 10^−11^ constitutes the most precise test of CPT invariance in the baryon sector. However, it is limited by magnetic field fluctuations imposed by the antiproton decelerator and ELENA-synchrotron operated in the AMF hall. Advancing the shielding of our superconducting magnet operated at a field of *B*_0_ = 1.945 T further is also challenging^19^.

Inspired by these limitations, and to markedly advance the limits of precision antiproton measurements, we propose a strategy that can lead to a new era of precision antimatter spectroscopy. Transporting antiprotons and their applications have been predicted and discussed earlier^20–23^, but only one study reported the relocation of electrons in a superconducting magnet in a closed trap chamber^24^. Our approach involves relocating antiprotons from the AMF of CERN using the transportable Penning-trap BASE-STEP to dedicated high-precision offline laboratories, in which advanced spectroscopy can be performed under disturbance-free conditions. For the next generation of antiproton precision measurements, we require that external magnetic field fluctuations contribute less than 1 nT uncertainty in frequency ratio measurements. In a 1-T magnetic field with a shielding factor of 100 (ref. ^19^) and averaging the centre to 10% of the linewidth, antiproton-based CPT tests with statistical uncertainty of the order of 10^−12^ become possible, corresponding to an improvement by more than one order of magnitude compared with the present state of the art.

In this study, we present a milestone towards the realization of antiproton transportation in the next years. Using the open Penning-trap system BASE-STEP, which can transfer the trapped particles into another experiment, we successfully transported a cloud of around 100 trapped protons out of the AMF of CERN and demonstrated lossless particle transport on a truck across the Meyrin campus of CERN. Within our 4-h transport campaign, the persistent superconducting magnet system operated autonomously, based on battery supplies, cryopumping and cooling by a liquid helium (LHe) reservoir. Following the transport, we returned the trap to its original location and continued the experimental operation. Subsequently, we demonstrated the ability to manipulate the transported particles, separating them into fractions and ejecting them from the trap. We thereby validated the feasibility of the concept to conduct antiproton precision studies offline. This crucial step towards improved measurements of the fundamental properties of the antiproton is also a demonstration of the movement of other accelerator-production-based charged particles into a cleaner measurement environment, for example, the exotic highly charged ions Pb^81+^ and U^91+^ (refs. ^13,14^) and other charged antimatter systems that may become available in the future, such as the antihydrogen ion ${\bar{{\rm{H}}}}^{+}$ (ref. ^11^) and the antihydrogen molecular ion ${\bar{{\rm{H}}}}_{2}^{-}$ (ref. ^12^).

**Fig. 1:**
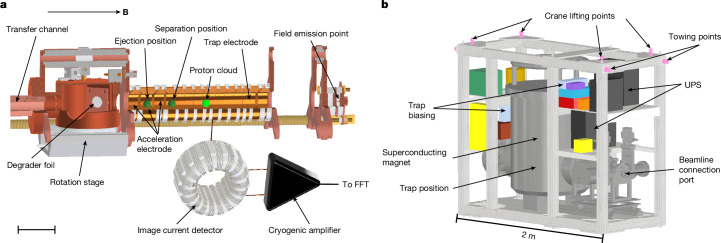
Experimental setup. **a**, Overview of the trap system components of the BASE-STEP apparatus used in this study. Parts of the full trap system^9^ have been omitted, and the field emission point was moved closer to the proton cloud for better visibility. A cloud of protons is created by an electron beam from the field emission point and stored inside the cylindrical electrodes of the trap. The image-current detector (which is used for non-destructively monitoring the trap content), the rotatable degrader stage and the transfer channel (which is used for ejecting into another trap system) are shown. **b**, A model of the transport frame containing the superconducting magnet with the trap system inside. The frame contains the UPS batteries, a control PC, frequency generators for particle manipulation and detection, voltage supplies for trap biasing, amplifiers and heaters.

The BASE-STEP trap system^9^ is shown in Fig. 1a. It is located in the horizontal cold bore (4 K) of the transportable superconducting magnet, which is shown in Fig. 1b. This system is connected to an antiproton transfer line with its beamline connection port. For the injection and ejection of particles, this open trap system has a transfer channel towards the connection port with a diameter of 6 mm and a length of 135 mm, acting at the same time as a pumping barrier. A cryogenic, rotatable trap electrode, a rotatable degrader stage^25^, and an inlet valve^9^ protect the trap vacuum from molecular background flow. Simulations indicate that at 4 K, with a closed inlet valve, background pressures of the order of 10^−16^ mbar can be reached^9^.

The trap consists of a stack of cylindrical, gold-plated electrodes, made from oxygen-free high conductivity copper, engineered in compensated and orthogonal design^26^. Voltages applied to the electrodes provide an electrostatic quadrupole potential that stores the particles along the trap axis, which is parallel to the magnetic field lines, leading to an axial oscillation with a frequency of *ν*_*z*_ ≈ 383.3 kHz at a central ring electrode voltage of −2.950 V. A superconducting image-current detection system for the non-destructive detection of the trapped protons and measurements of their axial frequency *ν*_*z*_ is connected to one of the trap electrodes^27^. Apart from the axial mode, two circular modes exist in the plane perpendicular to the magnetic field lines. With a magnetic field *B*_0_ between 136 mT and 1.005 T, the modified cyclotron frequency *ν*_+_ is between 2.02 MHz and 15.14 MHz, and the magnetron frequency *ν*_−_ is between 40 kHz and 5 kHz, respectively.

The transportable trap system is integrated into the frame as shown in Fig. 1b. It weighs between 850 kg and 900 kg and contains all devices necessary for transportation. The entire system is designed for transport on public roads, which includes a carbon-steel vacuum chamber for magnetic stray field shielding and a support structure for the cold mass of the magnet and the transport frame to handle acceleration forces apart from gravity of up to 1.0*g* in all directions^9^. When connected to the power grid, the cooling to cryogenic temperatures to operate the system is provided by a pulse-tube cooler, whereas in autonomous transport mode, the system is cooled by an internal 30 l LHe tank. The precision voltage supply necessary for biasing the trap electrodes, as well as frequency generators for particle manipulation, a spectrum analyser for non-destructive detection and a sensor array for transport monitoring are powered by an uninterruptible power supply (UPS) with two battery units, also mounted on the frame. Although this design keeps the transport system on a compact footprint of 2 m × 0.85 m, it requires the disconnection and reconnection of the system to the pulse-tube compressor to be handled within a limited timeframe. Before moving the system, we verified, in several test runs, that the LHe and the UPS battery capacity are sufficient to run autonomously for 4 h and that restarting the pulse-tube cooler does not quench the persistent current that is loaded in the superconducting magnet.

For the transport, protons are loaded into the Penning-trap system using an in-trap cryogenic field emission point that is operated with electron currents up to 150 nA. The current evaporates any adsorbates hitting the surfaces of the trap system and creates ions of a few species (p, ${{\rm{H}}}_{2}^{+}$, …) in the centre of the trap (Fig. 1a). Using the procedures described in ref. ^5^, we prepare a clean cloud of protons using stored-wave-inverse-Fourier-transform radiofrequency drives to remove contaminant ions. Then, the radial modes of the proton cloud are cooled using sideband cooling^28^. The number *N* of trapped particles prepared in this loading and cleaning sequence can be determined non-destructively, using the dip signature that is created by thermal-equilibrium particle–detector interaction^29^. The width of the dip Δ*ν* is given as

1 $$\Delta \nu =\frac{1}{2{\rm{\pi }}}\frac{N{q}^{2}{R}_{{\rm{p}}}}{m{D}^{2}}.$$

Here, *R*_p_ ≈ 80 MΩ is the effective parallel resistance of the detector, and *D* ≈ 0.0133 m is a trap-specific length. The amount of loaded particles can be adjusted based on the loading time and the applied field emission current. Depending on the experiments that are being conducted, we usually prepare 10–200 particles (Methods). For the transport that is reported here, we loaded *N* = 105(2) protons into the trap.

Although the particle loading takes place at *B*_0_ = 1 T, for the transport, the magnetic field is lowered to 136 mT. The reduction in magnetic field strength reduces the energy stored in the magnet and decreases the strength of the eddy currents induced during transportation. The low *B*_0_ also increases the critical temperature of NbTi by about 250 mK (ref. ^30^). Therefore, lowering the magnetic field reduces the risk and consequences of quenching the magnet, which is particularly important, because we encounter temperature spikes when reconnecting and restarting the pulse-tube cooler^9^ (Methods).

**Fig. 2:**
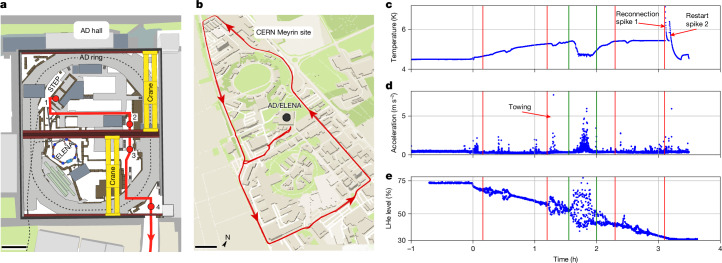
Transport route and sensor data. **a**, The route for the first transport demonstration through the AMF hall. Point 1 is the experiment zone from which an overhead crane moved the transport frame to point 2; at point 2, the transport frame was loaded onto a trailer and moved to point 3, where it then got picked up by the second overhead crane. Point 4 is the loading bay with the truck. **b**, Road map of the Meyrin site of CERN and the GPS position data recorded during transportation. Map reproduced from https://www.openstreetmaps.org. **c**–**e**, Magnet temperature (**c**), total acceleration (**d**) and liquid helium level (**e**) measured during transport. The zero on the horizontal axis marks the shutdown time of the pulse-tube cooler. For details, see the text and the Methods. The green bars enclose the time interval corresponding to the truck transport of the trap. The red bars indicate the start and stop times of the crane transportation.

As the next step, we switch all instruments to UPS operation, stop the pulse-tube cooler and disconnect the compressor from the pulse-tube unit. This marks the starting point to move the trap system within the AMF hall, as indicated by the red arrows and numbers in Fig. 2a. To transport the experiment along these paths, we use both overhead cranes in the AMF hall, moving BASE-STEP from points 1 to 2 and 3 to 4. Between 2 and 3, an industrial four-wheel flatbed trailer is used to transfer the instrument between the hooking points of the two cranes. At point 4, the trap system is lowered with the overhead crane onto a truck, towed and checked for radiation contamination before leaving the radiation-controlled area of the AMF hall. Then, the system is moved along the roads of the Meyrin site of CERN. In the last step, the trap system is craned back to the experiment area (point 4 to point 1), and the pulse-tube cooler is reconnected and restarted to continue the non-destructive operation of the experiment.

The GPS data recorded during the transport are shown in Fig. 2b, covering a distance of 3.72 km. In our attempt, a maximum velocity of 42.2 km h^−1^ was reached. The development of the magnet temperature, acceleration and LHe level recorded during the transport are shown in Fig. 2c–e. The two temperature spikes that occur when reconnecting the pulse-tube cooler and when its operation restarts pose a particular challenge. We have reduced the amplitudes of these temperature spikes by cooling the first stage of the pulse-tube cooler through a heat exchanger of the LHe tank exhaust and by tuning the power of a heater located on the LHe tank (Methods). Under these conditions, we observe 7.1 K and 6.4 K as peak values for the temperature spikes and maintain the persistent current in the superconducting magnet. In the future, the first spike could be eliminated by integrating the compressor of the cooler into the transport frame at the cost of increasing the size and weight of the system. Tilting the magnet frame also creates turbulence in the helium tank, as shown by the helium-level meter reading, resulting in a temporary cooling effect of the magnet. The acceleration data show its largest value of 7.7 m s^−^^2^ during the towing to the truck and up to 6 m s^−^^2^ while the truck was moving.

**Fig. 3:**
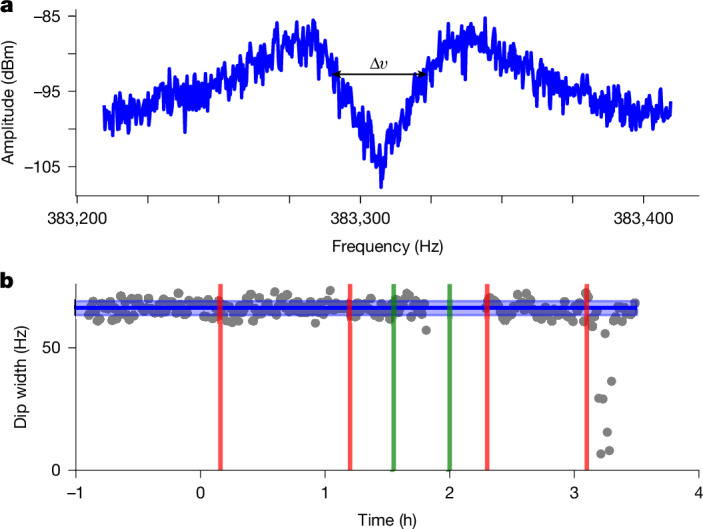
Particle detection during transport. **a**, FFT spectrum of the image-current signal showing the thermal noise of the superconducting LC circuit shorted by the trapped proton cloud at their axial frequency *ν*_*z*_ (ref. ^29^). **b**, Plot of the fitted dip width of the proton cloud during transport. The blue line with the surrounding faint blue bar is the mean value of the proton cloud before the transport, with its 1*σ* uncertainty to visualize the efficiency of the transport. The vertical red and green bars in **b** indicate the transport on the crane and the truck, respectively.

Throughout the entire transport, axial detector fast Fourier transformation (FFT) spectra are recorded while the axial frequency of the particles is tuned to the resonance frequency of the detector. A representative dip signal of the stored proton cloud is shown in Fig. 3a. The continuous monitoring of the protons during transportation is documented in Fig. 3b. Here the dip width *Δν* of the trapped protons is plotted as a function of time. During the transport on the truck, the particle and detector frequencies shifted because of a potential shift caused by the bad ground connection on one of the room-temperature filter boards for the electrode voltages and a movement of the semi-rigid detection wire in the trap, respectively. We needed about 15 min to match the axial frequency to the detector frequency and apply the magnetron sideband to exclude that the frequency shift is because of a sudden change in magnetron radius. This resulted in a lack of data close to the 2-h mark. This can be improved with improved soldering connections and additional fixtures on the trap wiring in the future. Furthermore, a reduction of the dip width is observed shortly after the 3-h mark, coinciding with the restart of the pulse-tube cooler. During this process, a temperature spike occurs, reducing the *Q*-value of the detector and consequently *R*_p_, which leads to less particle damping and thus smaller *Δν* (equation (1)). However, given the recorded data, it can be concluded that the transportation was lossless within the uncertainty of the measurement, and we were not able to detect any indication of heating of the circular *ν*_+_ and *ν*_−_ modes within the resolution limits of the measurement.

**Fig. 4:**
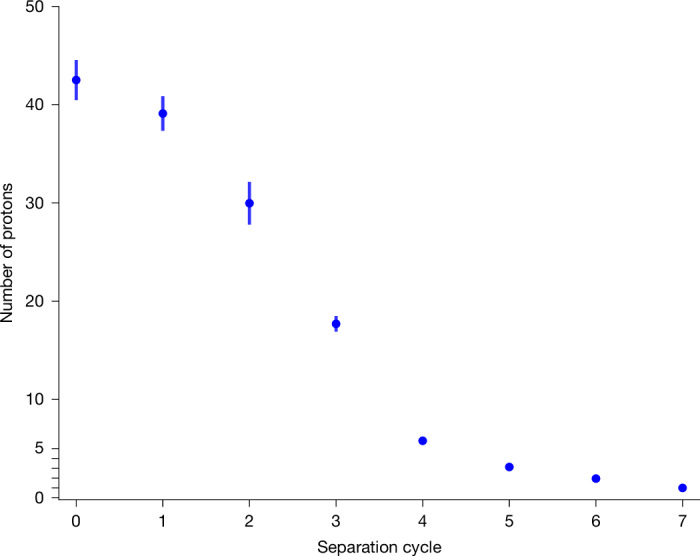
Proton cloud separation. Separation measurement to demonstrate that we can separate small fractions down to single protons from the stored cloud for the ejection procedure. The vertical lines indicate 1*σ* measurement uncertainties.

In our future planned antiproton transport experiments, the last step in the transport campaign would be the extraction of a fraction of particles from the trapped antiproton reservoir, followed by the injection of the extracted fraction into a receiver experiment. Although we do not have such a receiver available yet, we have demonstrated particle separation and extraction after returning to the experiment zone. This is shown in Fig. 4, which shows the number of protons as a function of a separation cycle executed in a similar way as in ref. ^31^. We split the proton cloud by voltage ramps and move the separated particles into the separation position (Fig. 1a) of the trap stack. Then, we eject them from the trap to mimic the transfer procedure. This protocol mitigates the risk of losing the entire reservoir during the transfer into a receiving precision-trap system, which is essential for the offline operation of antiproton experiments. Although the number of stored particles was not pushed to the limit, the transported number would already be enough for our high-precision experiments to operate for several years^32^. As an example, the non-destructive high-precision measurements performed in BASE at CERN typically consume around six antiprotons per year.

In summary, we have shown all key techniques to transport trapped antiprotons in the open Penning-trap system BASE-STEP to dedicated precision laboratories. Laboratories reachable within the 4-h autonomous operation time of BASE-STEP, provided by the LHe tank and the UPS units, can be supplied with the methods presented here. For long-distance transports, the compressor and a chiller needed for cooling the compressor will also be moved on the truck. This equipment requires 15 kW of power compared with the 150 W required to run the trap system. Therefore, this makes an onboard generator^10^ necessary as well. However, this setup ultimately enables the system to be continuously operated on roads across Europe without time restrictions.

Once the antiproton transport has been demonstrated, we are planning to deliver the particles to an offline state-of-the-art Penning-trap system now under construction at the Heinrich Heine University Düsseldorf, Germany, in which proton–antiproton CPT tests^33,34^ with an at least 100-fold improved precision will be performed. After the successful demonstration, parallelized measurements in multiple laboratories^35–37^ might become possible in the long term. Furthermore, an expansion of the antiproton offline program will require multiplication of transportable traps and a reduction of costs per device. Our transport demonstration at 136 mT implicitly shows the feasibility of particle injection after catching into transportable permanent-magnet traps, as developed in our laboratories at present. Established concepts for these devices^38^ will be adapted to the extremely high vacuum conditions for antimatter storage and transportation. This concept will also eliminate the operational risks of quenching the superconducting magnet and will enable a cost-efficient multiplication of transportable traps. Applying this technology to charged antimatter systems that will become available in the future—such as the antihydrogen ion ${\bar{{\rm{H}}}}^{+}$ (ref. ^39^) and the antihydrogen molecular ion ${\bar{{\rm{H}}}}_{2}^{-}$ (ref. ^40^)—will ensure that the predicted measurement precision by leveraging advanced experimental concepts^41^ is unaffected by the accelerator-induced magnetic field ramps. Thus, the developments demonstrated in this paper have the potential to pave the way towards a new era of precision physics with charged antimatter systems.

## Methods

### Transport setup

To ensure transportability, the space, mass and power consumption constraints outlined in the main text must be met. Consequently, only devices essential to primary functions are integrated into the transport frame shown in Fig. 1b, whereas non-essential components are disconnected before transport and remain in the experimental zone. This setup, termed the transport configuration, comprises four device groups.

The Core Systems are important for confining particles within the trap, primarily consisting of the high-precision voltage supply and a 750-Wh UPS supported by two lead-acid batteries. The voltage supply connects to the three electrodes of the trap centre, which confine the particle cloud, whereas all other electrodes are grounded. The magnet is self-sustaining in persistent mode and operates without active control. A LHe heater supports the cooling of the first stage of the heat shield of the magnet (see sections ‘Transport preparation and procedure’ and ‘Sensor data recorded during transport’). As shown in Extended Data Table 1, the experiment requires 150 W of power in transport configuration, allowing up to 5 h of off-grid operation. The battery life can be extended by reconnecting the UPS to power sockets in the vicinity during waiting times and the truck preparation. In this attempt, only 51% of the battery capacity was being used on return to the experimental zone during the transport described above.

Monitoring and control systems are implemented to monitor the proton cloud and sensor data, as well as to interact with the particles when necessary. To monitor the protons, the signal from the image-current detection system is processed using an FFT spectrum analyser. Conventional FFT analysers are bulky, weigh 25 kg and require a power consumption of up to 70 W. To get around these drawbacks, a custom FFT analyser based on an external soundcard, 1/50th the size and weight, was developed for this experiment. The detector signal is downmixed to the frequency range of the soundcard using waveform generator 1 (Extended Data Table 1). The soundcard records the signal and transmits it to the transport control personal computer (PC), a mini PC on the frame. This PC then analyses the sampled signal and generates FFT spectra, from which the dip width and, consequently, the number of protons can be extracted, as shown in the section ‘Particle number calibration’. Moreover, the PC collects and shows real-time sensor data alongside the recorded spectra, and it runs a basic trap control program to adjust electrode voltages. It also enables us to cool the radial modes of the proton through sideband coupling^28^. The radiofrequencies for cooling are supplied by waveform generator 2 (Excitation), which stays in standby mode to conserve power until required. A router is used to establish a wireless local network, enabling remote control and monitoring of the experiment via a laptop or smartphone.

The vacuum systems comprise the vacuum chambers and connected pumps. The experiment includes two separate vacuum systems, the magnet vacuum chamber and the inlet vacuum chamber^9^. The first one is the insulation vacuum of the magnet, which is pumped out by a permanently installed turbomolecular pump (TMP), and a backing scroll pump behind a valve. The inlet chamber vacuum is connected to the cryogenic trap chamber and defines the time for the monolayer formation inside the trap chamber (see main text). It is pumped by two TMPs in series that are permanently installed and can be shut off by a gate valve. Their backing scroll pump is removable for transport. Moreover, a non-evaporable getter pump (NEG) is installed at the inlet chamber.

Sensors are connected to the transport control PC to gather information about the trap status during transport as shown in Fig. 2 and discussed in detail in section ‘Sensor data recorded during transport’. Six temperature sensors track the magnet and heat shield temperatures, whereas two pressure gauges monitor vacuum conditions in the magnet and inlet chambers. A LHe level meter records helium consumption, and a microcontroller-based telemetry logger tracks the acceleration on the transport frame. It captures linear and angular accelerations, as well as magnetic field variations along three axes. The logger also includes a GPS antenna for position data.

### Transport preparation and procedure

In preparation of the transport, the LHe tank is filled, and all devices required for trap operation and data acquisition are put into transport configuration, see section ‘Transport setup’ for reference. During the transport, the valve connections of all TMPs are closed, and the pumps are shut down because there is no power available for their operation. The prepumps are disconnected and remain in the experiment zone. The NEG pump continues to operate as it requires no external power. A spreader beam is attached to the four lifting point connections on the transport frame to keep it horizontal at typically less than 1° inclination during the craning phase. With the crane ready, the time-critical part of the transport is started by activating the helium heater with a power of 2 W to boil off LHe and compensate for the heat load on the first stage of the pulse-tube cooler during transport. Subsequently, the helium recovery line is disconnected, and a non-return valve emits the evaporated helium into the atmosphere. About 13.5 l of liquid helium are lost during the transport sequence. As the next step, the pulse-tube cooler is stopped, setting the zero-hour reference in the plots of Fig. 2. With the shutdown of the pulse-tube cooler, the magnet is only cooled by the helium inside the tank, setting the time limit of the transport to 4 h. Then, the LHe level becomes too low, and there is a risk of quenching the magnet during the restart of the pulse-tube cooler or when starting to fill LHe.

The flex lines of the pulse-tube cooler are disconnected, and the pressure build-up in the pulse tubes is suppressed during transport because of the cooling by the LHe and heat exchange with the boiled-off gas. As a last action, the UPS power cord is removed, and the devices on the transport frame start to draw power from the UPS batteries. With the transport frame completely disconnected from the zone, the transport starts, and the path through the AMF hall and over the street is shown in Fig. 2a.

After the return of the transport frame to the zone, first the UPS system was reconnected to the power grid, and then the pulse-tube cooler was reconnected to the compressor and restarted. The helium heater operation continued until the LHe tank pressure was higher than the recovery line pressure to avoid heating of the experiment by rushing warm gas into the helium tank. Subsequently, the heater was shut off after the helium recovery line was reconnected and the pumps were restarted, finishing the transport and reconnection procedure.

### Sensor data recorded during transport

The most relevant sensor data recorded is shown in Fig. 2, in which GPS data, temperature, total acceleration and the LHe level are shown. The time zero is referenced to the shutdown time of the pulse-tube cooler, and the timing of the other events is shown in Extended Data Table 2.

With the stop of the pulse-tube cooler, the data in Fig. 2c show an increase in temperature on the magnet as the pulse-tube cooler starts to warm up. During operation, it actively cools the system, but when shut off, the pulse tubes conduct heat to the magnet, which is thermally linked with copper heat conductors to both the pulse-tube cooler and the LHe tank. This shifts the equilibrium temperature of the magnet, resulting in levelling of the temperature curve to about 5.5 K. The temperature spikes because of the reconnection of the flexlines of the pulse-tube coolers, and its restart occurs at the end of the transport procedure (see main text). In both cases, the helium gas in the pulse-tube cooler is set into motion, from equalizing the pressure of the compressor and the pulse-tube cooler, and from starting the compressor, respectively. To counteract both, we need to decrease the temperature on the heat shield connected to the first stage of the pulse-tube cooler, which is not directly connected to a liquid reservoir for cooling. We use the heater element located on the LHe tank to apply 2 W heating power to evaporate more helium and cool the heat shield by the heat exchanger in the exhaust of the helium tank. With this, we achieve a heat shield temperature increase from 51 K to only 66 K during the transport. After the restart of the pulse-tube cooler, the magnet starts to cool to its initial temperature of about 4.5 K.

The temperature curve in Fig. 2b also shows four indents, most noticeable during the truck transport. Driving on the road creates turbulence in the LHe tank over a longer time period, effectively reducing the temperature by 0.82 K to about 4.7 K temporarily. Similar behaviour can be seen at least three more times on the curve. All three of the smaller indents are a result of setting down the trap, in which the settling results in a small turbulence in the tank. The indent at  about 30 min is a result of setting the magnet down on the trailer to switch the overhead cranes (Fig. 2a) at point 2, whereas the indent at  around 1 h 20 min is the arrival in the loading bay (Fig. 2a) at point 4. At  about 2 h 25 min, the small indent is also a result of placing the trap on the trailer to change the overhead cranes. The total acceleration in Fig. 2d shows that these events align with moments of larger acceleration and also moments of turbulence in Fig. 2e, in which the LHe level in the tank is plotted. The acceleration on the transport frame mostly relates to events of rotation and tilting, creating turbulence in the LHe, which gets the liquid in contact with parts of the tank that are typically above the level of the liquid. At the top of the helium tank, a recondenser is installed with a strong thermal coupling to the pulse-tube cooler interface. Therefore, the motion of the liquid in the helium tank results in cooling the surfaces near or on the recondenser, counteracting the heat flow through the cryocooler, which results in the observed temporary cooling effect. Further supporting this explanation is the observation of the cooling to 4.7 K for roughly 20 min during the truck transport, which is the longest period of turbulence by rotation and tilting, whereas the events of large linear acceleration do not align with the strongest temporary cooling.

The largest acceleration event reached a recorded value of 7.7 m s^−2^, with the gyroscope data showing a rapid rolling motion by up to 2°. This is because of the towing procedure of the frame to the truck as listed in Extended Data Table 2. The trap is secured according to DIN EN 12195, in which the normal force between the cargo and the floor of the cargo area is increased by pulling the cargo onto the floor through the four towing lines and increasing the friction coefficient by placing a rubber pad below the cargo. During the settling, the force on the lines was not increased simultaneously, resulting in an uncontrolled motion of the frame, which is avoidable if the tows are tightened symmetrically. Smaller acceleration values are observed during rotation around the vertical axis on the crane, for example, when the lashing straps between the frame and the spreader beam are tightened. The craning operation produces only low acceleration, even for the lifting and settling on the ground. The acceleration data also show the arrival back in the experimentation zone and the reconnection process of the pulse-tube cooler flexlines (last entries in Extended Data Table 2).

The helium level in the magnet is kept constant during stationary operation because of the pulse-tube cooler operation. Without a heater, the standing time is about 12 h after switching the cryocooler off^9^. The helium evaporation starts as soon as the heating power of 2 W is applied to cool the heat shield, and we used about 45% (13.5 l) during the transport sequence with a 3 h and 21 min duration. We have characterized the pulse-tube cooler spikes for standing times of up to 4 h duration without transportation, showing that the magnetic field remains constant. Further increasing the maximum transport time is feasible; however, it requires time-consuming tests.

Regarding the vacuum conditions, we observe pressure spikes up to 10^−7^ mbar level in the magnet vacuum chamber during transport, which we attribute to spontaneous gas release from the multilayer insulation (MLI) foil around the different thermal stages of the magnet. The inlet chamber is free of MLI foil and does not show this behaviour; it remained at a constant pressure in the low 10^−8^ mbar range.

### Particle number calibration

**Extended Data Fig. 1:**
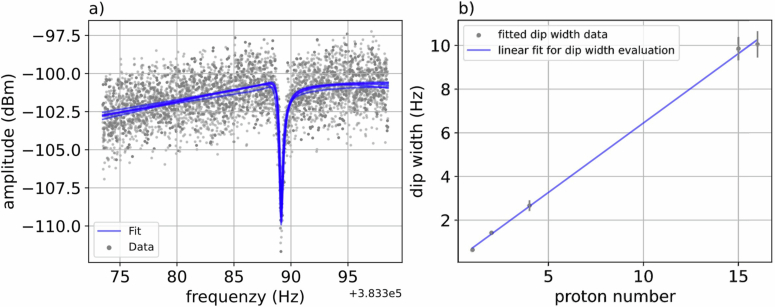
Plots for the dip width evaluation. a) Fitting results for a singe proton. In gray are the FFT data of 5 spectra, in blue the corresponding fit functions, resulting in a single proton dip width of (0.64 ± 0.06) Hz. b) Results of an evaporation measurement for dip width calibration, plotting the dip width against the corresponding proton number. In gray the data, in blue a linear fit for future data evaluation with (0.63 ± 0.01) Hz ⋅ *x* + (0.09 ± 0.13) Hz. The single proton value was taken from the results of graph a).

As shown in equation (1), the dip width on the resonator is linearly dependent on the number of trapped particles. To calibrate the slope, we, therefore, loaded a cloud of protons into the trap and started to evaporate protons in different step sizes from it. We fit the FFT spectrum using the appropriate line shape^29^ and assign the best-fitting particle numbers to each measurement by using the dip width of the single proton as a reference (Extended Data Fig. 1a). We apply a linear fit to the results to get a calibration, resulting in a single proton dip width on the resonator of 0.64 ± 0.06 Hz and a linear fit of (0.63 ± 0.01) Hz × *N* + (0.09 ± 0.13) Hz in Extended Data Fig. 1b), where *N* is the number of protons.

Using this calibration, the dip width data in Fig. 3b can be converted into particle number. Uncertainties in *N* arise from the calibration uncertainty (1.6%), systematic uncertainties due to the temperature and magnetic field dependence of *R*_p_ (1 %) and the statistical uncertainty. We determine 66.3(1.3) Hz and 65.6(1.3) Hz dip widths before and after the transport, corresponding to 105(2) and 104(2) protons, respectively. The values are consistent with a lossless transport as they agree within 0.4*σ* of their error bars.

### Particle loss mechanisms

Potential loss mechanisms of trapped protons that we deem important during transport are collisions or charge exchange reactions with residual gas, and radial expansion of large particle clouds. Charge exchange reactions lead to immediate particle loss, which was not observed. The other two mechanisms induce a growth of the magnetron radius of the trapped particles, and we can detect an axial frequency shift as a function of the magnetron radius because of the field imperfections of the trap before losing the particles. However, this was also not observed when storing the clouds for several days in the trap, and also not during transportation.

## Online content

Any methods, additional references, Nature Portfolio reporting summaries, source data, extended data, supplementary information, acknowledgements, peer review information; details of author contributions and competing interests; and statements of data and code availability are available at 10.1038/s41586-025-08926-y.

## Figures and Tables

**Extended Data Table 1:**
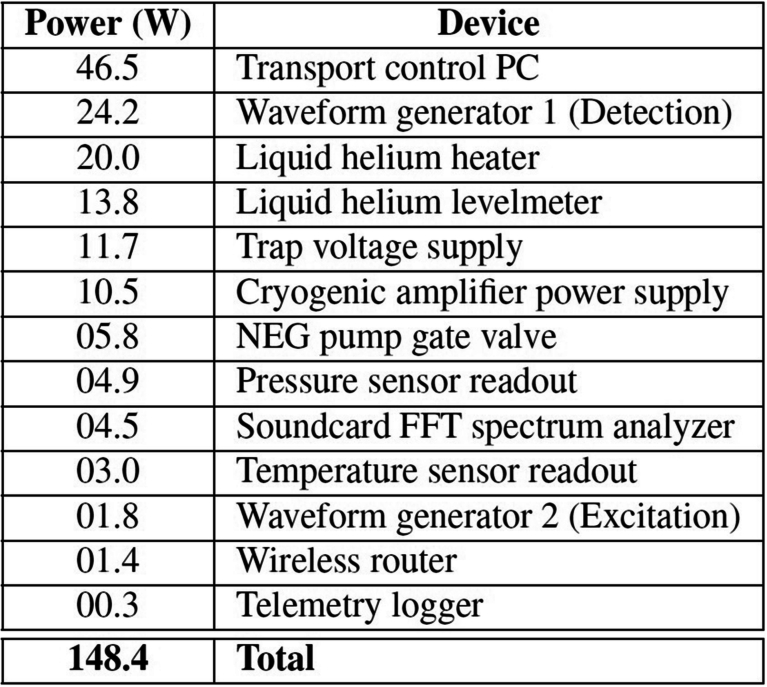
Power consumption

The average power consumption in transport configuration broken down by devices from highest consumption to lowest.

**Extended Data Table 2:**
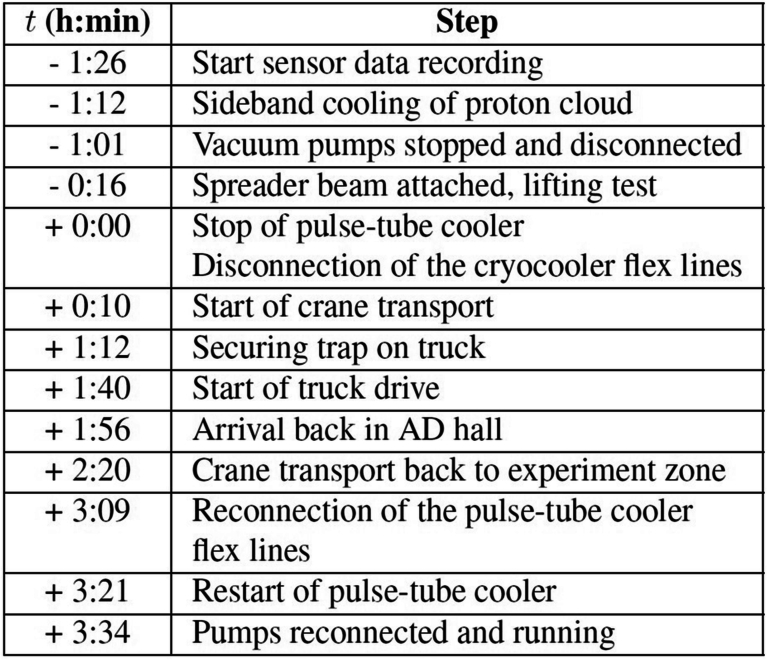
Timetable of the transport procedure

The time *t* is given in (hours : minutes). The table starts with the start of data recording by various sensors like magnet temperature and acceleration. The shut down of the pulse-tube cooler and the start of the disconnection of the flex lines between the compressor and the cooler is the reference mark for all times given in the table.

## Data Availability

The data sets will be made available on reasonable request.

## Code availability

Analysis codes will be made available on reasonable request.

## References

[1] Bartmann, W. et al. The ELENA facility. *Philos. Trans. R. Soc. A Math. Phys. Eng. Sci.* **376**, 20170266 (2018). 10.1098/rsta.2017.0266 PMC5829171 29459416

[2] Hori, M. & Walz, J. Physics at CERN’s antiproton decelerator. *Prog. Part. Nucl. Phys.* **72**, 206–253 (2013).

[3] Hori, M. et al. Buffer-gas cooling of antiprotonic helium to 1.5 to 1.7 K, and antiproton-to–electron mass ratio. *Science* **354**, 610–614 (2016). 27811273 10.1126/science.aaf6702

[4] Ahmadi, M. et al. Characterization of the 1S–2S transition in antihydrogen. *Nature* **557**, 71–75 (2018). 29618820 10.1038/s41586-018-0017-2 PMC6784861

[5] Smorra, C. et al. BASE – the baryon antibaryon symmetry experiment. *Eur. Phys. J. Spec. Top.* **224**, 3055–3108 (2015).

[6] Smorra, C. et al. A parts-per-billion measurement of the antiproton magnetic moment. *Nature* **550**, 371–374 (2017). 29052625 10.1038/nature24048

[7] Schneider, G. et al. Double-trap measurement of the proton magnetic moment at 0.3 parts per billion precision. *Science* **358**, 1081–1084 (2017). 29170238 10.1126/science.aan0207

[8] Borchert, M. et al. A 16-parts-per-trillion measurement of the antiproton-to-proton charge-mass ratio. *Nature* **601**, 53–57 (2022). 34987217 10.1038/s41586-021-04203-w

[9] Smorra, C. et al. BASE-STEP: a transportable antiproton reservoir for fundamental interaction studies. *Rev. Sci. Instrum.* **94**, 113201 (2023). 37972020 10.1063/5.0155492

[10] Aumann, T. et al. PUMA, antiproton unstable matter annihilation: PUMA collaboration. *Eur. Phys. J. A* **58**, 88 (2022).

[11] Pérez, P. et al. The GBAR antimatter gravity experiment. *Hyperfine Interact.* **233**, 21–27 (2015).

[12] Myers, E. G. CPT tests with the antihydrogen molecular ion. *Phys. Rev. A* **98**, 010101 (2018).

[13] Kluge, H.-J. et al. in *Current Trends in Atomic Physics*, Vol. 53 of *Advances in Quantum Chemistry* (eds Salomonson, S. & Lindroth, E.) 83–98 (Academic Press, 2008).

[14] Sturm, S. et al. The ALPHATRAP experiment. *Eur. Phys. J. Spec. Top.* **227**, 1425–1491 (2019).

[15] Safronova, M. S. et al. Search for new physics with atoms and molecules. *Rev. Mod. Phys.* **90**, 025008 (2018).

[16] Bertone, G., Hooper, D. & Silk, J. Particle dark matter: evidence, candidates and constraints. *Phys. Rep.* **405**, 279–390 (2005).

[17] Dine, M. & Kusenko, A. Origin of the matter-antimatter asymmetry. *Rev. Mod. Phys.* **76**, 1–30 (2003).

[18] Anderson, E. K. et al. Observation of the effect of gravity on the motion of antimatter. *Nature* **621**, 716–722 (2023). 37758891 10.1038/s41586-023-06527-1 PMC10533407

[19] Devlin, J. A. et al. Superconducting solenoid system with adjustable shielding factor for precision measurements of the properties of the antiproton. *Phys. Rev. Appl.* **12**, 044012 (2019).

[20] Dehmelt, H. Economic synthesis and precision spectroscopy of anti-molecular hydrogen ions in Paul trap. *Phys. Scr.* **1995**, 423 (1995).

[21] Lewis, R., Smith, G. & Howe, S. Antiproton portable traps and medical applications. *Hyperfine Interact.* **109**, 155–164 (1997).

[22] Martin, J. et al. Overview of the high performance antiproton (HiPAT) experiment. In *Proc.**17th International Conference on the Application of Accelerators*, 20020091877 (NASA, 2002).

[23] Wada, M. & Yamazaki, Y. Technical developments toward antiprotonic atoms for nuclear structure studies of radioactive nuclei. *Nucl. Instrum. Methods Phys. Res. B Beam Interact. Mater. Atoms* **214**, 196–200 (2004).

[24] Tseng, C. H. & Gabrielse, G. Portable trap carries particles 5000 kilometers. *Hyperfine Interact.* **76**, 381–386 (1993).

[25] Latacz, B. M. et al. Ultra-thin polymer foil cryogenic window for antiproton deceleration and storage. *Rev. Sci. Instrum.* **94**, 103310 (2023). 10.1063/5.0167262 37874231

[26] Gabrielse, G., Haarsma, L. & Rolston, S. Open-endcap Penning traps for high precision experiments. *Int. J. Mass Spectrom. Ion Process.* **88**, 319–332 (1989).

[27] Nagahama, H. et al. Highly sensitive superconducting circuits at ∼700 kHz with tunable quality factors for image-current detection of single trapped antiprotons. *Rev. Sci. Instrum.* **87**, 113305 (2016). 27910537 10.1063/1.4967493

[28] Cornell, E. A., Weisskoff, R. M., Boyce, K. R. & Pritchard, D. E. Mode coupling in a Penning trap: *π* pulses and a classical avoided crossing. *Phys. Rev. A* **41**, 312–315 (1990). 9902871 10.1103/physreva.41.312

[29] Wineland, D. J. & Dehmelt, H. G. Principles of the stored ion calorimeter. *J. Appl. Phys.* **46**, 919–930 (1975).

[30] Bottura, L. *A Practical Fit for the Critical Surface of NbTi.* Report No. CERN LHC Report 358 (European Organization for Nuclear Research, 1999).

[31] Smorra, C. et al. A reservoir trap for antiprotons. *Int. J. Mass Spectr.* **389**, 10–13 (2015).

[32] Sellner, S. et al. Improved limit on the directly measured antiproton lifetime. *New J. Phys.* **19**, 083023 (2017).

[33] Ding, Y. & Kostelecký, V. A. Lorentz-violating spinor electrodynamics and Penning traps. *Phys. Rev. D* **94**, 056008 (2016).

[34] Ding, Y. Lorentz and CPT tests using Penning traps. *Symmetry* **11**, 1220 (2019).

[35] Bohman, M. et al. Sympathetic cooling of a trapped proton mediated by an LC circuit. *Nature* **596**, 514–518 (2021). 34433946 10.1038/s41586-021-03784-w PMC8387233

[36] Cornejo, J. M. et al. Quantum logic inspired techniques for spacetime-symmetry tests with (anti-)protons. *New J. Phys.* **23**, 073045 (2021).

[37] Schüssler, R. X. et al. Detection of metastable electronic states by Penning trap mass spectrometry. *Nature* **581**, 42–46 (2020). 32376960 10.1038/s41586-020-2221-0

[38] Gomer, V., Strauss, H. & Meschede, D. A compact Penning trap for light ions. *Appl. Phys. B* **60**, 89–94 (1995).

[39] Perez, P. & Sacquin, Y. The GBAR experiment: gravitational behaviour of antihydrogen at rest. *Class. Quantum Grav.* **29**, 184008 (2012).

[40] Myers, E. G. The most precise atomic mass measurements in Penning traps. *Int. J. Mass Spectr.* **349–350**, 107–122 (2013).

[41] Cornejo, J. M. et al. Resolved-sideband cooling of a single ^9^Be^+^ ion in a cryogenic multi-Penning-trap for discrete symmetry tests with (anti-)protons. *Phys. Rev. Res.* **6**, 033233 (2024).

